# Krüpple-like-factor 4 Attenuates Lung Fibrosis via Inhibiting Epithelial-mesenchymal Transition

**DOI:** 10.1038/s41598-017-14602-7

**Published:** 2017-11-20

**Authors:** Lianjun Lin, Qian Han, Yan Xiong, Ting Li, Zhonghui Liu, Huiying Xu, Yanping Wu, Nanping Wang, Xinmin Liu

**Affiliations:** 1 The Geriatrics Department, Peking University First Hospital, Beijing, China; 2 The Pathology Department, Peking University First Hospital, Beijing, China; 3 Key Laboratory of Molecular Cardiovascular Science of Ministry of Education, Peking University Health Science Center, Beijing, China

## Abstract

Epithelial-mesenchymal transition (EMT) plays an important role in the pathogenesis of idiopathic pulmonary fibrosis (IPF). Krüpple-like-factor 4 (KLF4), has been suggested to play an important role in the phenotype transition. However, its function in pulmonary fibrosis and EMT of human alveolar epithelial cells (AECs) remains unclear. This study aimed to examine the role of KLF4 in pulmonary fibrosis and EMT. Decreased expression of KLF4 was first observed in human IPF lung tissues and models of bleomycin-induced pulmonary fibrosis. Transgenic mice with overexpression of KLF4 were subjected to bleomycin-induced pulmonary fibrosis model and showed attenuated lung fibrosis and EMT compared to wild type group. Furthermore, the effects overexpression and knockdown of KLF4 on TGF-β1-induced EMT were examined in AECs. Adenovirus-mediated overexpression of KLF4 attenuated TGF-β1-induced EMT and activation of Smad2/3 and Dvl in AECs. Conversely, knockdown of KLF4 promoted the activation of pathways above mentioned and TGF-β1-induced EMT. Our results demonstrates that KLF4 plays an important role in bleomycin-induced lung fibrosis through suppressing TGFβ1-induced EMT. Thus, it may serve as a potential target for the treatment of pulmonary fibrosis.

## Introduction

Idiopathic pulmonary fibrosis (IPF), a progressive and lethal disease with poor prognosis, lacks effective therapy strategies^1^. Although the pathogenesis of IPF remains poorly understood, it is widely accepted that aberrant injury-repair of epithelial cells plays a role in IPF^2,3^. The formation of fibrotic foci is a pathological hallmark of IPF in which activated fibroblasts and myofibroblasts are believed to be the main effector cells by secreting extracellular matrix proteins and undergoing structure remodeling^4–6^. Of the several potential origins of activated fibroblasts and myofibroblasts, injured epithelial cells contribute to the formation of pulmonary fibrosis through epithelial-mesenchymal transition (EMT)^7–9^.

EMT is a process in which fully differentiated epithelial cells are transformed into a mesenchymal phenotype, with the loss of epithelial markers, acquisition of mesenchymal property, reassembly of cytoskeleton with enhanced migratory. EMT is involved in embryogenesis physiologically and carcinogenesis pathologically^10^. Moreover, EMT has been implicated in the pathogenesis of organ fibrosis such as kidney^11^ and lung^8,9,12,13^. There is evidence of EMT in fibroblastic foci in IPF, indicating that EMT is involved in fibrosis development^8,9,13^. Furthermore, approximately one third of fibroblasts are of epithelial origin in bleomycin-induced pulmonary fibrosis^12^. Of multiple stimuli involved in lung fibrosis, TGF-β1 is an important pro-fibrotic factor that has been shown to induce EMT both *in vitro* and *in vivo* ^8,10,14^ and signaling pathways either through the canonical pathway involving downstream phosphorylation of Smad 2/3 or through other non-Smad pathways such as Wnt/β-catenin pathway^15^.

Krüpple-like family is a group of transcription factors containing of evolutionarily conserved zinc-finger DNA binding domain^16,17^ and regulates diverse cell processes^18^. A family of 17 mammalian KLFs have been discovered^19,20^. Among them, KLF4 performs multiple functions in a number of physiological and pathological processes. Recent studies have shown that KLF4 expression is decreased in lung cancer^21^ and pulmonary arterial hypertension^22^. However, its expression in lung fibrosis is not known. A recent study demonstrated that KLF4 was down-regulated in TGF-β-induced EMT in human renal proximal tubule epithelial cells (HK-2 cells) and in mice subjected to unilateral ureteral obstruction (UUO) which is a model of kidney fibrosis^23^. KLF4 inhibited EMT in renal epithelial cells^23^, hepatocellular carcinoma cells^24^, and mouse lung epithelial LA-4 cells^25^. However, the expression and function of KLF4 in pulmonary fibrosis and EMT of human lung alveolar epithelial cells (AECs) remains unclear. Thus, we hypothesized that epithelial KLF4 may modulate EMT and lung fibrosis.

In this study, we demonstrated that the expression of KLF4 was decreased in lung tissues of human IPF and mouse models of bleomycin-induced pulmonary fibrosis. Overexpressing of KLF4 inhibited bleomycin-induced pulmonary fibrosis and EMT *in vivo* and attenuate TGF-β1-induced EMT in AECs *in vitro*. These results provide novel evidence that KLF4 represents a potential therapeutic target in IPF.

Some of the results of this study have been previously reported in the form of an abstract^26^.

## Results

### The Expression of KLF4 Was Decreased in Fibrotic Tissues of IPF Patients and mouse models of Bleomycin-induced pulmonary fibrosis

**Figure 1:**
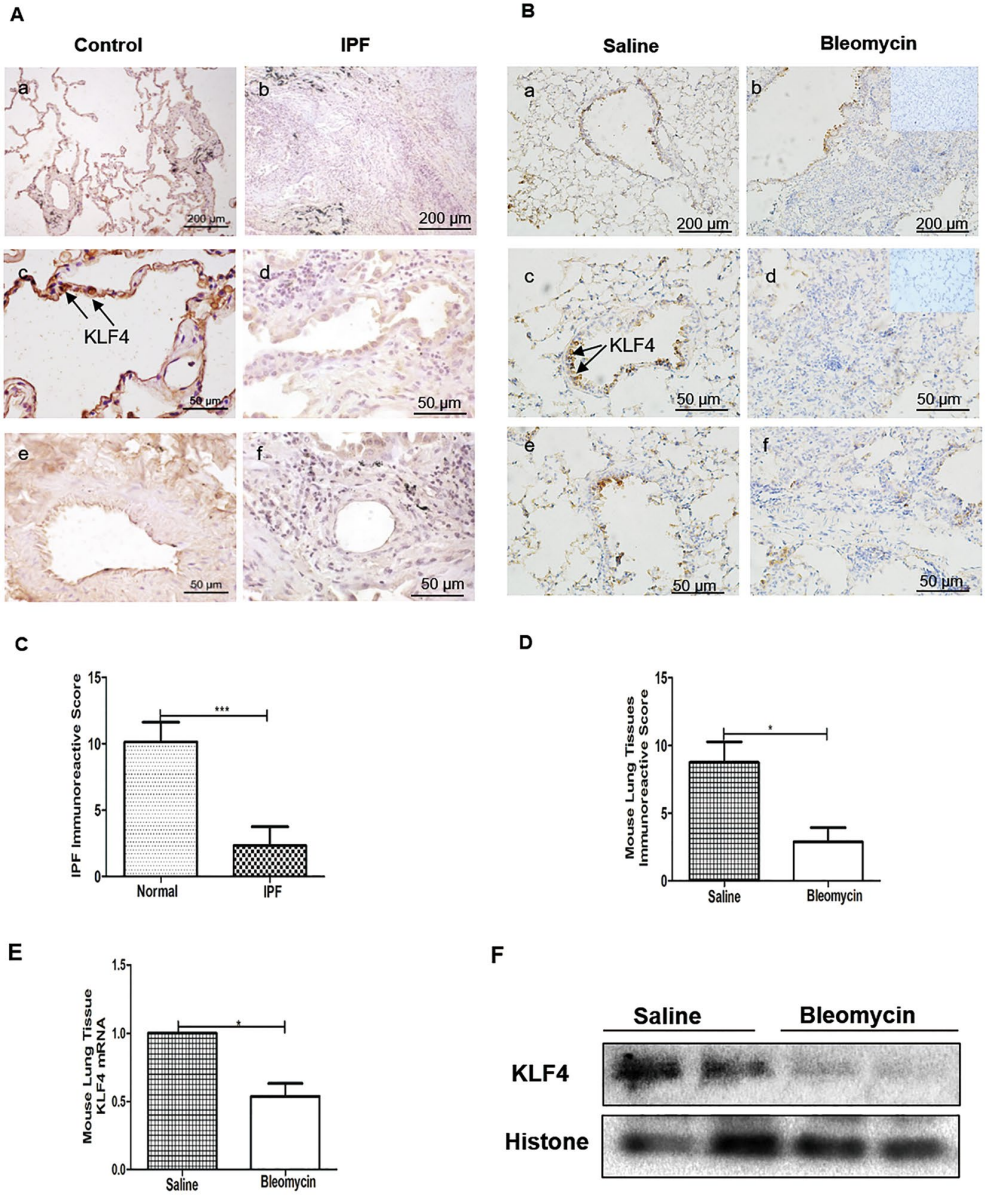
KLF4 expression was downregulated in human IPF lung tissues and mouse models of bleomycin-induced pulmonary fibrosis. Representative microphotographs of immunohistochemistry staining of KLF4 in cancer adjacent normal lung tissue (**A**, left column) and fibrotic tissues (**A**, right column) of IPF patients, and mouse models of bleomycin-induced pulmonary fibrosis (**B**, right column) or control saline group (**B**, left column). KLF4 expression level in sections of IPF patients or control group (**C**), and bleomycin-induced fibrosis model or control group (**D**) were shown by immunoreactive system (IRS) and analyzed.Scale bar was as indicated in the figure. qPCR and western blotting were performed to evaluate the expression of KLF4 in mouse lung tissues of bleomycin-induced pulmonary fibrosis and control group (**E,F**). The values were expressed as mean ± SD. N = 4 for each group. ***P < 0.001 by *t*-test.

To determine KLF4 expression in IPF, lung tissue sections from 5 IPF patients and 5 lung cancer adjacent normal tissues were acquired to undergo immunohistochemistry staining. IPF biopsy samples exhibited typical hallmarks of IPF with temporal and spatial heterogeneity and the presence of fibrotic foci (Fig. 1A,b). In normal lung tissues, KLF4 was mainly expressed in the nucleus and cytoplasm in alveoli epithelial cells, endothelial cells and epithelium of bronchiole (Fig. 1Aa,c,e). In contrast, in lung biopsies from IPF patients, expression of KLF4 was significantly decreased, especially in the more severe fibrotic tissue. Only sporadic expressions of KLF4 in nucleus or cytoplasm of epithelium of bronchiole and endothelial cells of vessel (Fig. 1A,d,f) were observed. The percentage and intensity of KLF4 expression in IPF lung tissue was prominently decreased (Fig. 1C).

**Figure 2:**
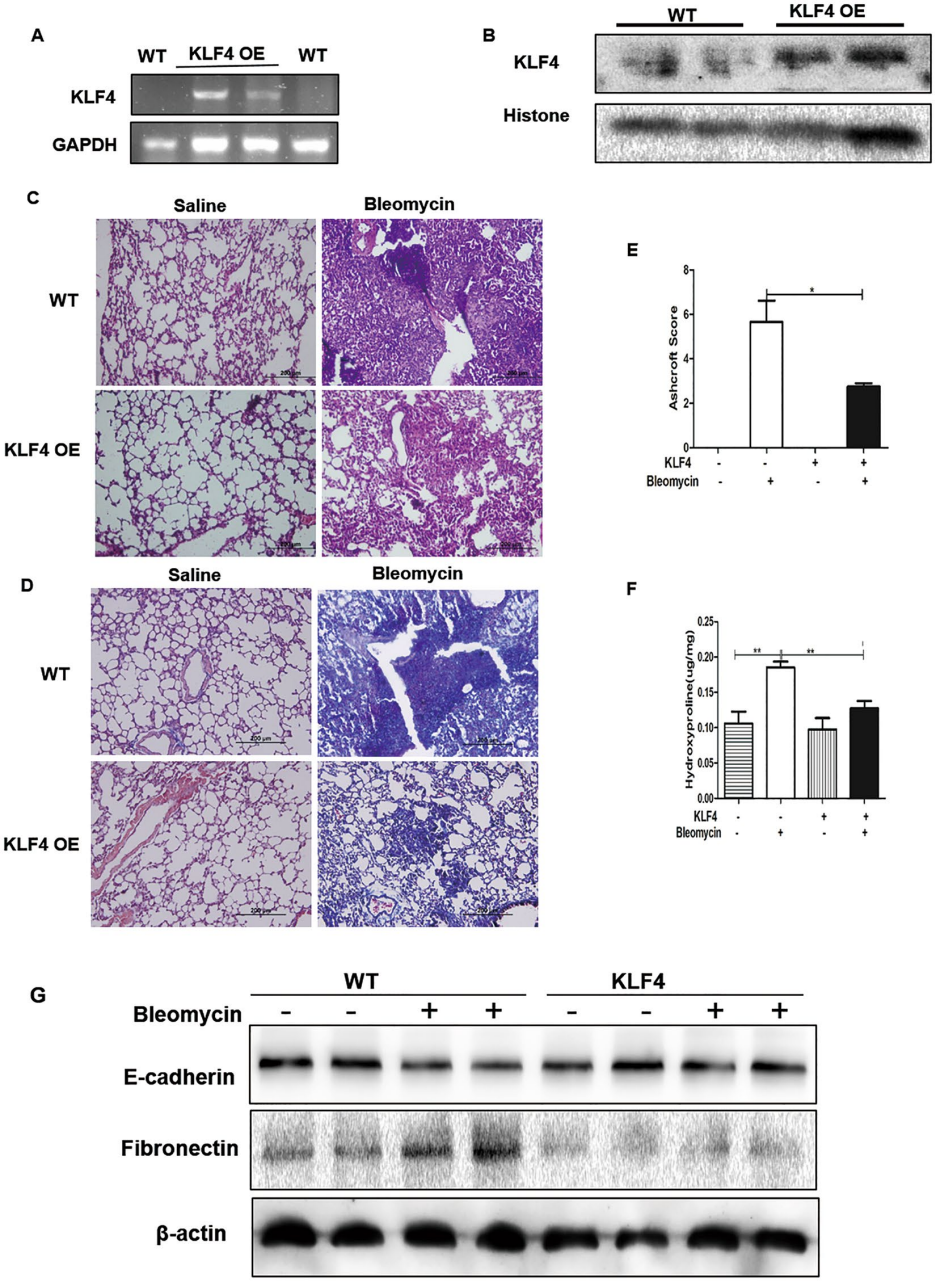
Overexpression of KLF4 inhibited bleomycin-induced pulmonary fibrosis *in vivo*. Overexpression of KLF4 in the transgenic mice were confirmed with PCR (**A**) and western-blotting (**B**). Mice were intratracheally intubated and injected with bleomycin to induce pulmonary fibrosis Saline was used as a control, in overexpression of KLF4 mice group and wild-type group. H&E staining (**C**) and Masson staining (**D**) were used to detect collagen depositions. Ashcroft scores showed the degree of fibrosis (**E**). The lung tissues of mice were harvested and assayed for hydroxyproline analysis (**F**). The expression of E-cadherin and fibronectin was evaluated by western blotting (**G**) in KLF4 overexpressing group and wild-type group. Representative images were from five mice per group. Scale bar = 200 μm. The values were expressed as mean ± SD from N = 5 in each group. *P < 0.05 and **P < 0.01 by *t*-test.

To further investigate the expression of KLF4 in pulmonary fibrosis (Fig. 1B,D), FVB mice were subjected to bleomycin-induced pulmonary fibrosis model (Fig. 2C,D). Consistent with the results in human lung tissues, the expression of KLF4 was mainly in nucleus and sporadically in cytoplasm in mouse lung tissues as well. It was mainly expressed in the epithelium of bronchus, alveoli epithelium, and endothelium (Fig. 1B). In bleomycin-induced pulmonary fibrosis, the expression of KLF4 was decreased, especially in the fibrotic area (Fig. 1B,b,d and f). KLF4 expression area and intensity was quantified by immunoreactive system (IRS) (Fig. 1D) and it showed that expression of KLF4 was decreased in bleomycin-induced pulmonary fibrosis, compared with controls. Moreover, qRT-PCR and western blotting were performed and confirmed that expression of KLF4 was down-regulated in bleomycin-induced pulmonary fibrosis tissues at both mRNA and protein levels (Fig. 1E,F).

### Overexpression of KLF4 Attenuated Lung Fibrosis in Bleomycin-induced Pulmonary Fibrosis Model

To investigate the effect of KLF4 on pulmonary fibrosis i*n vivo*, transgenic mice with overexpression of KLF4 were constructed. The expression of KLF4 at mRNA and protein levels was observed to be significantly up-regulated (Fig. 2A,B). The transgenic mice was administrated with intratracheal instillation of bleomycin in both KLF4-overexpressing mice and wild-type group. Severe lung fibrosis was present in bleomycin-administered wild-type mice (Fig. 2C). The severity of lung fibrosis and collagen fiber accumulation were decreased in KLF4-overexpressing transgenic mice as shown by H&E, Masson staining, the Aschoff score and hydroxyproline analysis (Fig. 2C–F). Additionally, the expression of E-cadherin was down-regulated and fibronectin was up-regulated in bleomycin-induced pulmonary fibrosis model, compared with the saline group (Fig. 2G). In KLF4-overexpressing group, the down-regulation of E-cadherin was attenuated and up-regulation of fibronection was inhibited, compared with wild-type group. These results suggested that EMT may be inhibited in the overexpressing KLF4 transgenic mice (Fig. 2G).

### Overexpression of KLF4 Attenuated EMT in Bleomycin-induced Pulmonary Fibrosis

**Figure 3:**
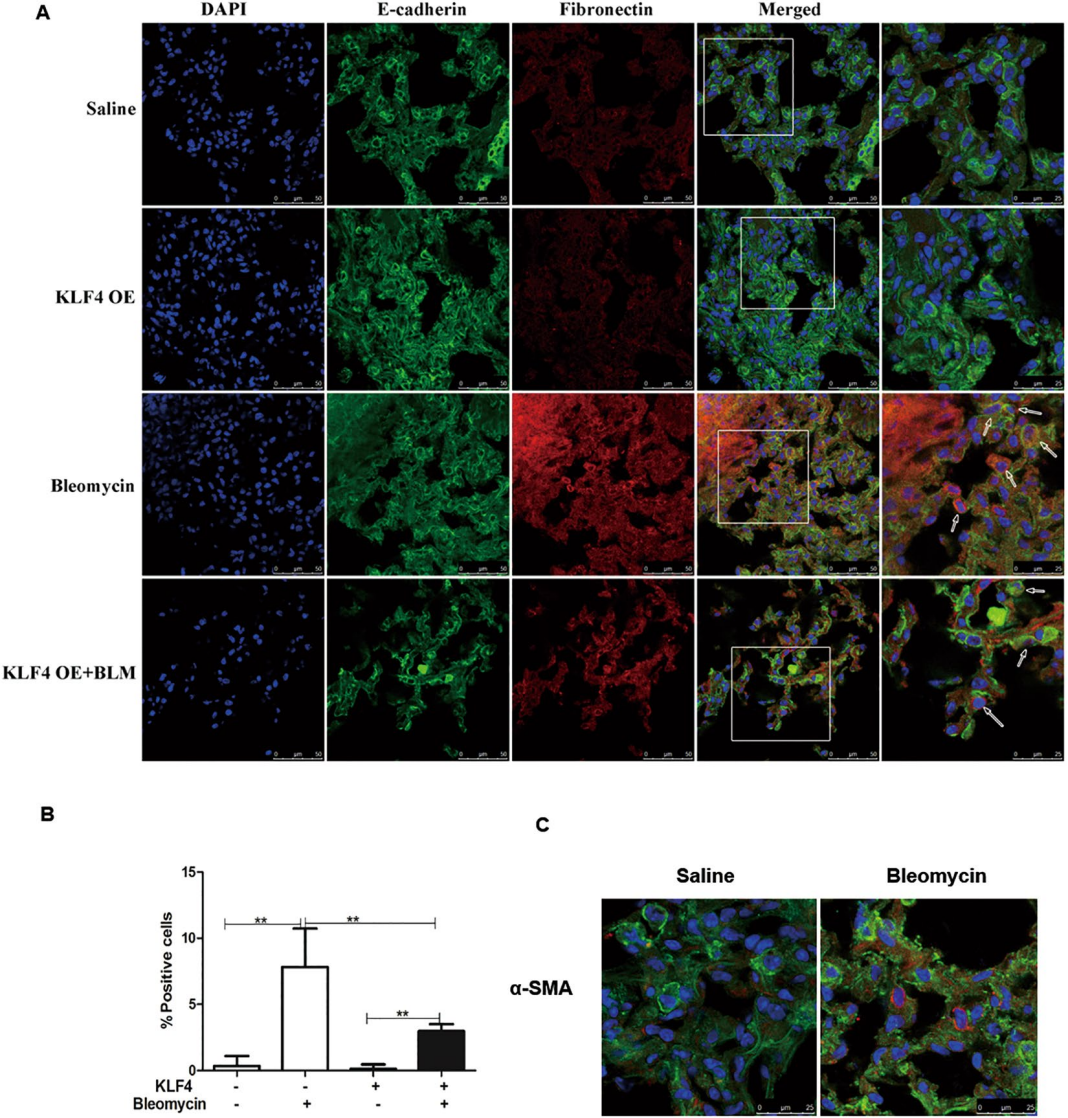
Overexpression of KLF4 attenuated EMT in bleomycin-induced lung fibrosis *in vivo*. Mice were intratracheally intubated and given bleomycin or saline as control to induce pulmonary fibrosis overexpression in KLF4 transgenic mice group and wild-type group. Double-labelled immunofluorescent staining were performed to examine the expression of E-cadherin (green) and fibronectin (red) as indicated by the white arrow. Scale bar = 50μm and 25μm as indicated in the figure (**A**). The percentage of double-label positive cells was calculated in each group (**B**). Double-labelled immunofluorescent staining was detected E-cadherin (green) and α-SMA (red). Scale bar = 25 μm (**C**). Representative image of three independent experiments were shown. The values were expressed as mean ± SD from five samples in each group were shown. **P < 0.01 by *t*-test.

To assess the presence and extent of EMT in bleomycin induced pulmonary fibrosis model, expression levels of E-cadherin and fibronectin were analyzed in sections of lung tissues from mice administrated with saline or bleomycin in wild-type group and KLF4-overexpressing transgenic group with double-label immunofluorescent staining method. Double stained cells were detected in the fibrotic areas (Fig. 3A). Very few double positive cells were observed in saline group (Fig. 3A). It is estimated that approximately 10% of cells undergoing EMT, characterized by positive staining of both epithelial and mesenchymal phenotype markers colocalized in the lungs from bleomycin-induced pulmonary fibrosis model. Overexpression of KLF4 significantly decreased the percentage of cells undergoing EMT in pulmonary fibrosis model (Fig. 3A,B). The proportion of cells undergoing EMT in lungs of bleomycin-induced pulmonary fibrosis model with overexpression of KLF4 was about 3.8%. Moreover, dual staining positive cells for E-cadherin and α-smooth muscle actin (α-SMA) were observed in bleomycin-induced pulmonary fibrosis (Fig. 3C), indicating that there were cells transited from an epithelial to myofibroblast phenotype in this model.

### Overexpression of KLF4 Attenuated TGF-β1-induced EMT in Alveolar Epithelial Cells

To further confirm the role of KLF4 in EMT of alveolar epithelial cells, we examined the effect of KLF4 on TGF-β1-induced EMT in AECs and A549 cells (Supplemental Figures).

**Figure 4:**
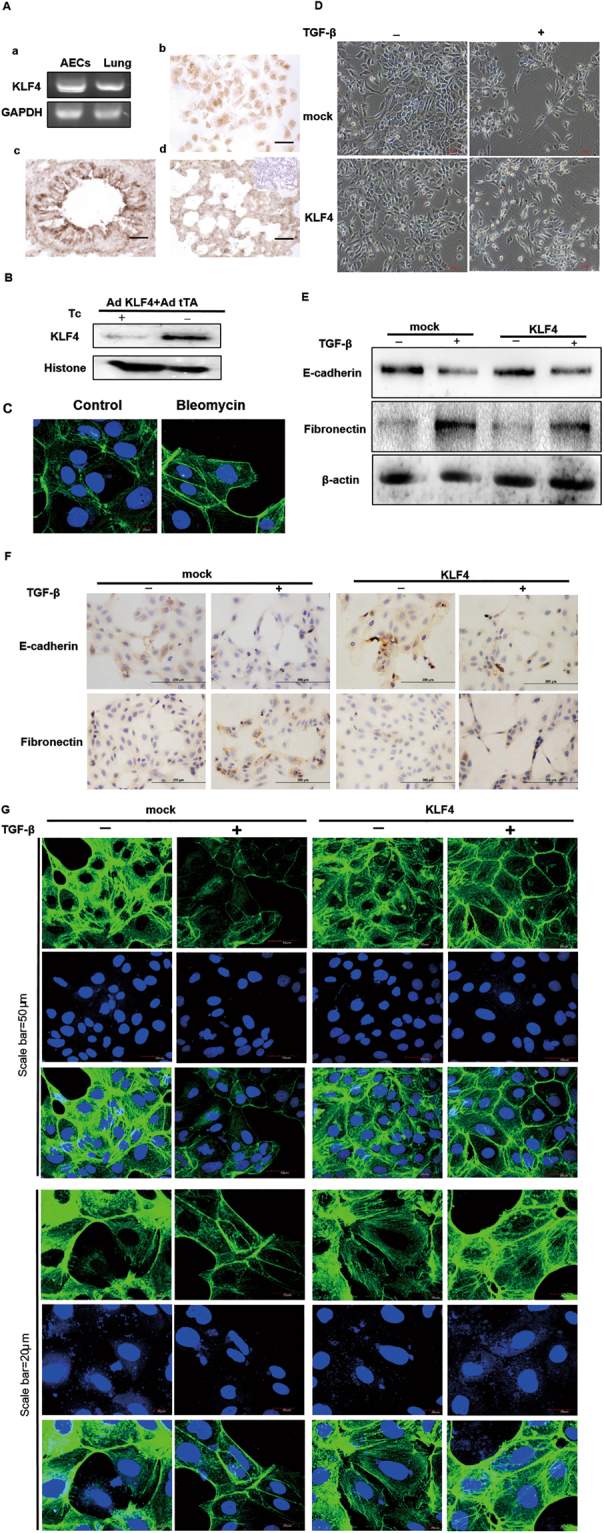
Overexpression of KLF4 attenuated TGF-β1-induced EMT of alveolar epithelial cells. (**A**) KLF4 mRNA level expression in AECs^(a)^ and lung tissues from mice^(b)^ was assessed with PCR. Immunocytochemistry was performed using a primary antibody against KLF4 to show the expression of KLF4 in AECs^(c)^ and lung tissue of mice^(d)^. Scale bar = 50 μm for (**A**). (**B**) AECs were co-infected with AdKLF4 and AdtTA (20 MOI) and maintained in the medium with or without tetracycline (Tc; 0.1 μg/mL). Nuclear protein lysates were immunoblotted with antibodies against KLF4 or Histone as an internal control. (**C**) F-actin staining was performed to show the morphological changes in AECs upon TGF-β1 stimulation. (**D**) AECs were co-infected with AdKLF4 and AdtTA in the medium with or without tetracycline and then treated with or without TGF-β1 (5 ng/mL) for 48 hours.The TGF-β1-stimulated morphological change in AECs with or without overexpression of KLF4 was observed under phase contrast light microscopy. (**E**) Total proteins were immunoblotted with antibodies against E-cadherin and fibronectin. β-actin was used as a loading control. Data shown are representative of three independent experiments. (**F**) Immunochemistry staining of E-cadherin and fibronectin in TGF-β1-induced EMT in AECs with and without overexpression of KLF4. (**G**) Cells were fixe F-actin staining (Green) was shown. Nuclei were counterstained with DAPI (Blue). ((**A**) Scale bar = 50 μm. (**B**) Scale bar = 20 μm). Data shown are representative of three independent experiments.

Firstly, we examined the expression of KLF4 in AECs and lung tissue samples from normal FVB mice by RT-PCR and immunohistochemistry staining. As shown in Fig. 4A, KLF4 was expressed mainly in the nucleus but also in the cytoplasm of AECs, epithelium of bronchiole and epithelium of alveoli of normal mouse lung tissues.

Secondly, to explore the effect of KLF4 on TGF-β1-induced EMT in AECs, we used a tetracycline-regulated adenovirus to overexpress KLF4 (AdKLF4). As shown in Fig. 4B, overexpression of KLF4 was observed in AECs upon infection of AdKLF4 and Ad tetracycline transactivator (AdtTA).

Thirdly, to examine whether induced expression of KLF4 could affect EMT, AECs were infected with AdKLF4 and AdtTA for 24 h and then stimulated with TGF-β1 for 48 h. TGF-β1 was chosen because of its widely accepted pro-fibrotic and EMT function in lung epithelial cells. TGF-β1-induced morphological change in AECs was shown in Fig. 4C by staining of F-actin. Western blotting and immunohistochemistry showed that overexpression of KLF4 markedly attenuated TGF-β1-induced downregulation of E-cadherin and upregulation of fibronectin (Fig. 4E,F). Besides, KLF4 inhibited the morphological change induced by TGF-β1 as shown in Fig. 4D by images taken under phase contrast light microscopy. During TGF-β1-induced EMT, overexpression of KLF4 preserved the cobble-like epithelial shape and reduced the spindle-shaped mesenchymal cell appearance of AECs.

To demonstrate EMT-associated cytoskeletal rearrangement, F-actin staining by phalloidin was performed. After stimulation of TGF-β1, reorganization of cortical filaments occurred. AECs acquired a more fibroblast-like morphology together with formation of elongated F-actin stress fibers. Overexpression of KLF4 preserved the cortical actin architectures in cells stimulated with TGF-β1 and reduced the incidence of elongated stress fiber formation (Fig. 4G).

### RNA Interference of KLF4 Enhanced TGF-β1-induced EMT in Alveolar Epithelial Cells

**Figure 5:**
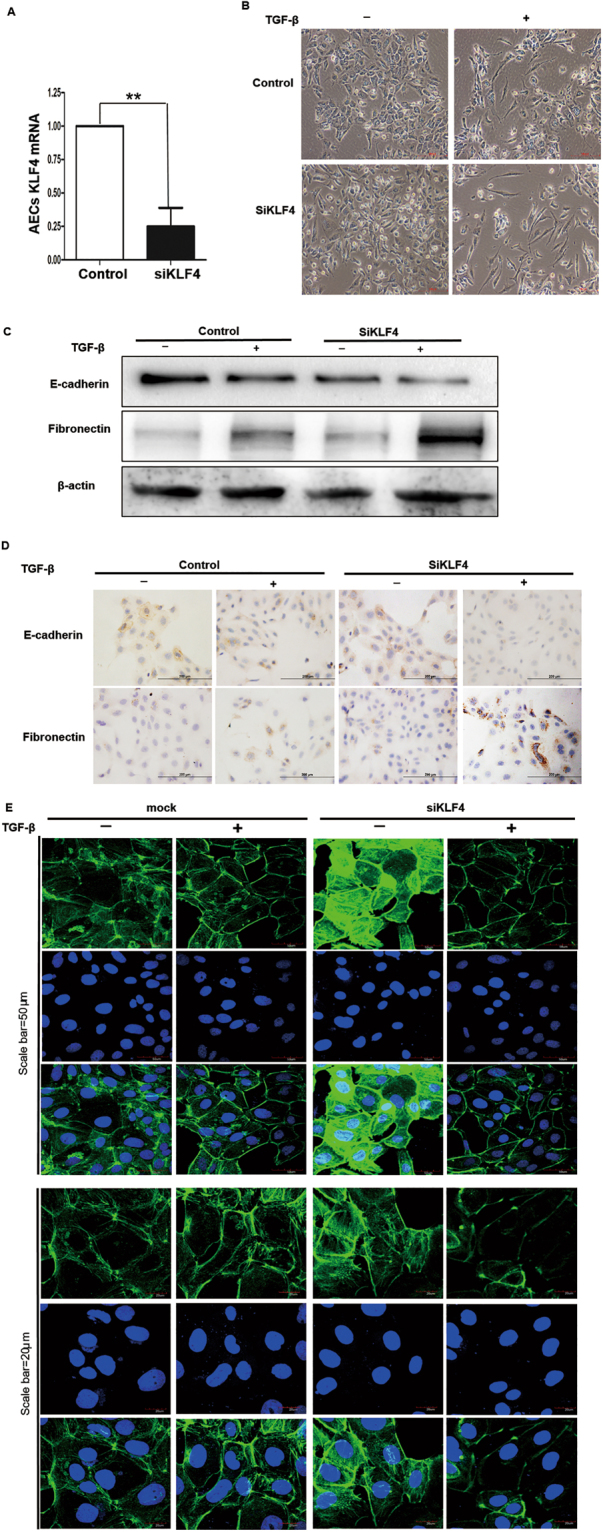
Knockdown of KLF4 promoted TGF-β1-induced EMT in alveolar epithelial cells. AECs were transfected with KLF4 siRNAs or control siRNA (100 nM) for 48 h. KLF4 expression level was assessed with qRT-PCR (**A**). AECs transfected with siRNA or control siRNA were stimulated with TGF-β1 for 48 hours. The TGF-β1 induced morphological changes were shown (**B**). Total proteins were immunoblotted with antibodies against E-cadherin and fibronectin. β-actin was used as loading control (**C**). Immunocytochemistry staining of E-cadherin and fibronectin in KLF4 knock down and control AECs (**D**). (**E**) RNA interference of KLF4 F-actin staining (Green) was shown. Nuclei were counterstained with DAPI. ((**A**) Scale bar = 50 μm. (**B**). Scale bar = 20 μm). Data shown are representative of three independent experiments.

To examine whether endogenous KLF4 plays a role for EMT in alveolar epithelial cell, we transfected AECs with KLF4 siRNAs or control siRNA. KLF4 siRNA was shown to efficiently decrease KLF4 mRNA level (Fig. 5A).

To elucidate the effect of KLF4 on TGF-β1-induced EMT in alveolar epithelial cells, AECs were transfected with KLF4 siRNAs or control siRNA for 48 h and stimulated with TGF-β1. Knockdown of KLF4 potentiated TGF-β1-induced EMT as shown by downregulation of E-cadherin and upregulation of fibronectin, compared to control siRNA (Fig. 5C,D). Moreover, knockdown of KLF4 also decreased endogenous expression of E-cadherin and slightly enhanced expression of fibronectin.

Furthermore, knockdown of KLF4 promoted the morphological change induced by TGF-β1 as shown in Fig. 5B by images taken under phase contrast light microscopy and Fig. 5E by F-actin staining. Knockdown of KLF4 promoted the morphological change as shown by more elongated spindle-like mesenchymal cells upon the stimulation of TGF-β1 (Fig. 5B,E). Meanwhile, knockdown of KLF4 enhanced the incidence of elongated stress fiber formation upon TGF-β1 stimulation (Fig. 5E).

### KLF4 Inhibited TGF-β1-induced Phosphorylation of Smad2/3 and Dvl Signaling in AECs

**Figure 6:**
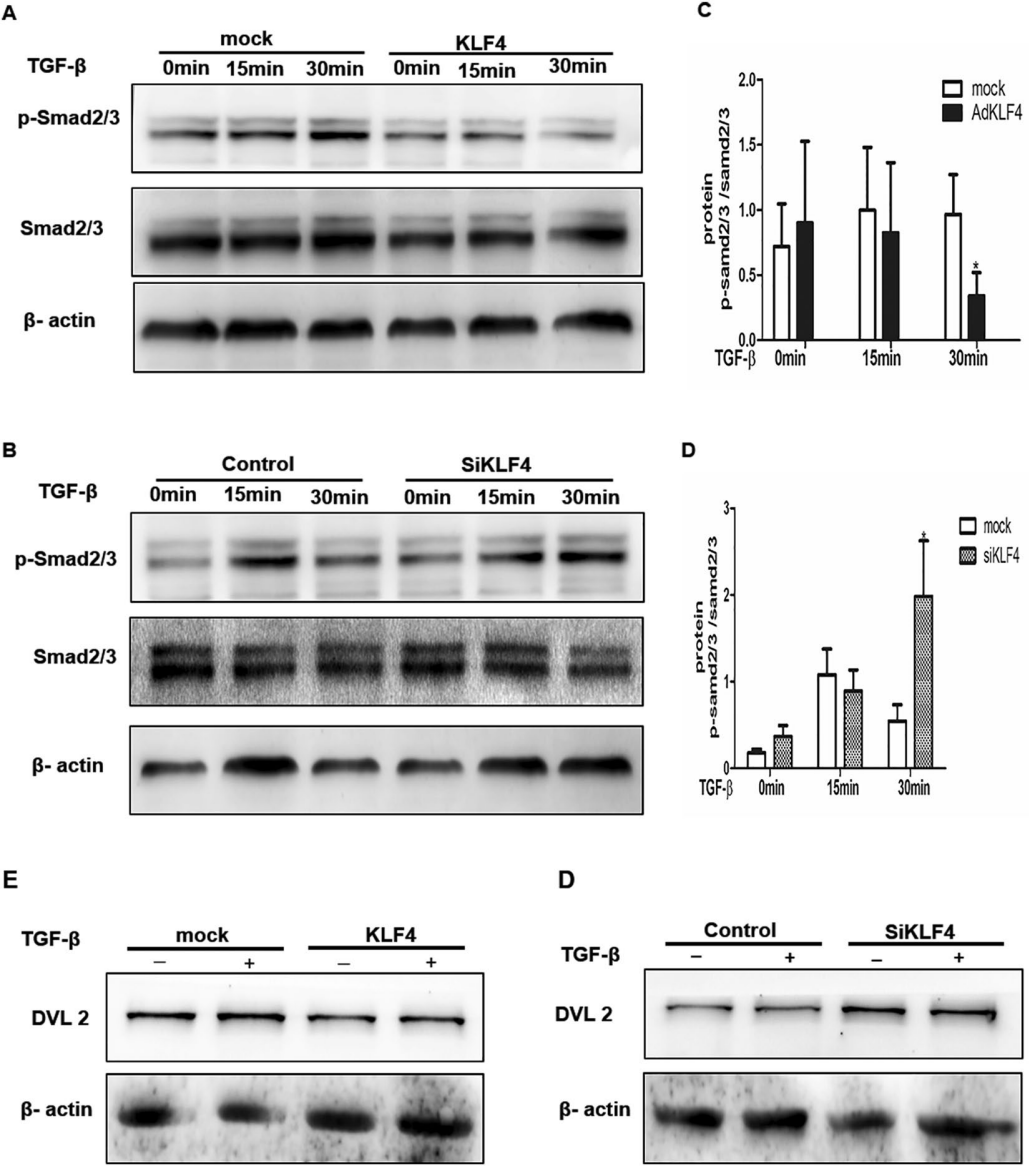
KLF4 inhibited TGF-β1-induced phosphorylation of Smad2/3 and Dvl. AECs were co-infected with AdKLF4 and AdtTA in the medium with or without tetracycline and then stimulated with or without TGF-β1 (5 ng/mL) for the indicated time (**A**,**E**). AECs were transfected with KLF4 siRNAs or control siRNA (100 nM) and then were stimulated with TGF-β1 (5 ng/mL) for indicated time (**B**,**F**). Total protein lysate was immunoblotted with antibodies against Smad2/3, their phosphorylated forms, DVL-2 and β-actin. Densitometry analysis of figure A and B was performed with imageJ (**C**,**D**). Similar results were obtained in three independent experiments.

To elucidate the signaling pathway through which KLF4 modulates TGF-β1-induced EMT, AECs were co-infected with AdKLF4 and AdtTA in the medium with or without tetracycline and then stimulated with TGF-β1 for the indicated times. Overexpression of KLF4 inhibited TGF-β1-induced phosphorylation of Smad2/3 in canonical TGF-β/Smad pathway (Fig. 6A) and decreased expression of Dvl2 in Wnt/β-catenin signaling pathway (Fig. 6C). On the contrary, knockdown of KLF4 potentiated TGF-β1-induced phosphorylation of Smad2/3 (Fig. 6B) and increased expression of Dvl2 (Fig. 6D). These results demonstrate that endogenous KLF4 play an important role in TGF-β1-induced EMT, probably by modulating the Smad and Wnt pathway.

## Discussion

In this study, we describe the novel finding that the expression of KLF4 was decreased in human IPF lung tissues and mouse models of bleomycin-induced pulmonary fibrosis, when compared to the corresponding controls. Our results showed that KLF4 attenuated bleomycin-induced pulmonary fibrosis and EMT *in vivo* and TGF-β1-induced EMT in AECs in vitro.

KLF4 is a zinc-finger transcription factor that plays an important role in cellular differentiation and proliferation during normal development and in various diseases. The expression of KLF4 is enriched in colon and moderate in distal small intestine, testis and lung tissues in adult mice at mRNA level^27^. As to lung, KLF4 is discovered to be the most significantly altered lung gene at birth and protein product was expressed in fibroblast and airway epithelial cells of perinatal lung tissues of mice^28^. KLF4 is also expressed in pulmonary arterial and venous endothelial cells in mice at mRNA level and protein level^29^. Pathophysiologically, lung KLF4 expression was reduced in PAH^22^, and lung cancer^21^. Here, our results show that the expression of KLF4 in surgical lung biopsy samples in normal controls and was decreased in IPF lung tissues. The expression of KLF4 was also observed in normal FVB mouse lung tissue and showed decreased level in bleomycin-induced pulmonary fibrosis model. Recently, Chen *et al*. showed that KLF4 was decreased in animal models of renal fibrosis^23,30^. Our data showed the first evidence that KLF4 is decreased in lungs of IPF patients and bleomcyin-induced pulmonary fibrosis.

The function of KLF4 in pulmonary fibrosis has not been previously described. Here, we report, for the first time, that KLF4 inhibited bleomycin-induced pulmonary fibrosis *in vivo*. Cowan *et al*. found that KLF4 depletion augmented lipopolysaccharide-induced lung injury and pulmonary edema *in vivo*, and concluded that KLF4 was important for VE-cadherin-mediated endothelial barrier function^31^. Shatat *et al*. found that knockdown of KLF4 exacerbated pulmonary hypertension in response to chronic hypoxia in mice^22^. These results indicated the importance of KLF4 in pulmonary function. Our data from transgenic mice with overexpression of KLF4 provide evidence to support KLF4 as a promising anti-fibrotic transcriptional factor.

EMT has been found to be involved in pathogenesis of fibrosis in many organs including lung. Here in our study, we demonstrated the presence of co-expression of epithelial marker and mesenchymal marker in bleomycin-induced pulmonary fibrosis shown by co-localization of E-cadherin and fibronectin, which suggests the existence of EMT. Moreover, results from semi-quantitative analysis of dual staining positive cells showed that overexpression of KLF4 inhibited the proportion of cells undergoing EMT in bleomycin-induced pulmonary fibrosis *in vivo*. Results from reporter-mice demonstrated that approximately one third of fibroblasts in lung fibrosis originated from epithelium^12^. Wu *et al*. confirmed airway EMT in BLM-induced peribronchial fibrosis mice^32^. However, Rock *et al*. concluded that no evidence of myofibroblasts were derived from Type II AEC^33^. There are some reasons that may lead to this inconsistent conclusion from different studies. Firstly, it should be kept in mind that EMT is a transient and dynamic state so it is really hard to capture the whole changing process *in vivo* by currently available methods. Secondly, as far as methods to elucidate EMT are concerned, co-localization by immune staining for EMT markers or the utilization of reporter mouse model, in different type of animal pulmonary fibrosis models or in human samples. Our data suggest that EMT does exist in bleomycin-induced pulmonary fibrosis. Moreover, KLF4 inhibited EMT in pulmonary fibrosis model *in vivo*.

As to the complexity and discrepancies of EMT *in vivo*, the results of studies about EMT *in vitro* are relatively definite and consistent. EMT is a process characterized by loss of epithelial markers such as E-cadherin, acquisition of mesenchymal markers such as α-SMA and fibronectin, change of morphology and reorganization of cytoskeleton. In pulmonary fibrosis, EMT is initiated by various types of pro-fibrotic stimuli to epithelium which lead to abnormal injury-repair. Our *in vitro* findings found that TGF-β1-induced EMT in AECs with the typical change of cellular morphology, phenotype marker transition, cytoskeleton rearrangements, and cell signaling pathway change, which were in consistence with results of other researchers. Overexpression of KLF4 attenuated EMT while knockdown of KLF4 promoted EMT with matched opposite tendency in both EMT-related phenotype and signaling pathways. Our data defined the function of KLF4 in EMT in human alveolar epithelial cells for the first time by overexpression and knockdown of KLF4. Moreover, KLF4 could maintain the expression of E-cadherin in AECs, which strengthens the important function of KLF4 in maintaining the epithelial phenotype.

One limitation of our study is that reason why KLF4 was down-regulated in pulmonary fibrosis still needs to be further investigated.

In conclusion, the present study demonstrates that the transcription factor KLF4 attenuates bleomycin-induced lung fibrosis and EMT *in vivo* and TGF-β1-induced EMT *in vitro*. Epithelial KLF4 is a promising potential target for further understanding the mechanism and developing novel strategy for the treatment of lung fibrosis in IPF and other EMT-related disease.

## Materials and Methods

### Patients

Five patients with IPF were included in this study and 5 cancer adjacent normal lung tissues were included as control. IPF diagnoses was dignosed by pulmonologists according to American Thoracic Society guideline^1^. These IPF patients had no history of cancer or other lung disease. The specimen sections were obtained from the Department of Pathology of Peking University First Hospital in Beijing, China. The study protocol was approved by the Ethics Committee of Peking University First Hospital and informed written consents were obtained from all participants involved in this study. All experiments were performed in accordance with relevant guidelines and regulations.

### Construction of Overexpression of KLF4 Mice

Mice with overexpression of KLF4 were constructed by injection of plasmid DNA of KLF4 into zygote of FVB mouse following standard pronuclear injection by Cyagen Biosciences (CA, USA). Positive founders were identified by PCR. Genotyping was done by PCR on toe DNA using the following primers: KLF4 (forward) 5′-CCGATGAACTGACCAGGCACTA, and (reverse) 5′-AGCGAGGAAGCGGAAGAGC. Wild-type littermates were used as controls. No difference in weight or survival rate was observed between overexpression group and wild type group. Mice were housed in a temperature- and humidity-controlled specific pathogen free facility, with standard chow and water ad libitum. Mouse were maintained on a 12 h light/12 h dark schedule at 22–25 °C with 45–65% humidity. All animal care and experimental procedures conformed to the Guide for the Care and Use of Laboratory Animals (NIH Publication no. 85-23, revised 1996) and were approved by the Animal Research Committee of Peking University First Hospital. All experiments were performed in accordance with relevant guidelines and regulations

### Bleomycin-induced Fibrosis Model

Male FVB mice with an average weight of approximately 25~30 g and aged from 8~11 weeks were intratracheally instilled with saline or 5 mg/kg of bleomycin on day 0 and 8. Mice were killed on day 21. Experiments were carried out using 5–7 mice per group. The lung tissues were collected for further analyses. Lungs were fixed in formalin and embedded in paraffin, sectioned and stained with hematoxylin and eosin (H&E) and Masson staining, or immunohistochemistry with antibodies against markers for EMT. Fibrosis was quantified using the whole lung by the Ashcroft scoring system. For immunofluorescent staining, lung tissues were embedded in Tissue-Tek OCT compound and snap frozen in liquid nitrogen. All experiments were performed in accordance with relevant guidelines and regulations.

### Cell Culture

Human primary alveolar epithelial cells (AECs) (Cell Biologics Inc, USA), were grown in Dulbecco’s modified Eagle’s medium (DMEM) supplemented with 10% fetal bovine serum (FBS), 0.1% Insulin-Transferrin-Selenium (ITS), 0.1% epithelial growth factor (EGF), 0.1% Hydrocortisone, 1% L-Glutamine, 2% Epithelial Cell Supplement and 1% Antibiotic-Antimycotic Solution at 37 °C in a humidified 5% CO2 atmosphere.

### Adenoviral Vectors and Infections

KLF4 adenovirus was constructed as previously described^34,35^. The expression of the inserted KLF4 was driven by a 7X tet operon/minimal cytomegalovirus promoter, that was further under the control of tetracycline-controlled transactivator (tTA). The adenoviruses were purified by cesium chloride methods. For adenovirus-mediated gene transfer, confluent AECs were exposed to adenoviral vectors with Ad-tTA to induce tetracycline controllable expression. Infected cells were incubated for the indicated time with or without tetracycline.

### Small-interfering RNA (siRNA)-mediated Gene Knockdown

The siRNA targeting human KLF4 mRNA (NM_004235) was synthesized with the sense sequence 5′-AGACGCUUCCAAGUUAUAU-3′. SiRNA for KLF4 and scrambled siRNA were purchased from Invitrogen (Carlsbad, CA). The double-strand RNAs (100 nM) were transfected into A549 cells with lipofectamine 2000 (Invitrogen). The control siRNA was used at the same dose.

### Western Blotting

Total proteins were extracted from AECs or mouse lung tissues with lysis buffer (50 mM Tris-HCL, pH 7.5, 15 mM EGTA, 100 mM NaCl, 0.1% Triton X-100 supplemented with protease inhibitor cocktail) and resolved on SDS-PAGE for electrophoresis. Nuclear proteins were prepared with the use of a high-salt buffer (20 mM Tris-HCL, pH 7.5, 15 mM MgCl2, 420 mM NaCl, 10% glycerol, 0.2 mM EGTA, supplemented with protease inhibitor cocktail). Immnunoblotting was performed with appropriate primary antibodies and a horseradish peroxidase (HRP)-conjugated secondary antibody followed by ECL detection (Amersham Biosciences, Fairfield, CT, USA).

### Quantitative Reverse Transcription-polymerasechain Reaction (qRT-PCR)

Total RNA was extracted from AECs or mouse lung tissues with Trizol reagent (Invitrogen, Grand Island, NY, USA). Two μg of total RNA was converted into cDNA with the use of reverse transcriptase and oligo (dT) (Promega, Madison, MI, USA) as a primer. Real-time quantitative PCR was performed using the iQ™ SYBR Green PCR Supermix in the DNA Engine Opticon realtime system (Bio-Rad Laboratories, Inc., Hercules, CA, USA) with GAPDH used as an internal control. The primer sequences are as follows: KLF4, 5′-AGGGGGTGACTGGAAGTTGT-3′ (forward), 5′-TTGCACATCTGAAACCACAG-3′ (reverse); GAPDH, 5′-ACCACAGTCCATGCCATCAC-3′ (forward), 5′-TCCACCACCCTGTTGCTGTA-3′ (reverse).

### Immunohistochemistry and Immunofluorescence

Lung tissues were fixed with 4% paraformaldehyde and embedded with paraffin. Cells were cultured on cover slips and subjected to staining. Sections were incubated with the appropriate primary antibodies at 37 °C for 1 h and 4 °C overnight. The secondary antibody was incubated for 1 h at 37 °C. Sections were viewed with light microscopy. Negative controls were performed by omitting the primary antibody.

As to phalloidin staining, F-actin was stained with FITC-conjugated phalloidin at a 1:1000 dilution in 2% BSA for at least 30 min. Hoechst was used to counterstain the nuclei. Images were acquired with confocal laser scanning microscope.

As to statistical analysis of double-staining positive cells, the confocal photos were taken under SP8 confocal microscopy (Germany) randomly and at least 5 visual fields were included in each section for further calculation. The number of cells and double-staining positive cells were manually counted and statistically analyzed.

To analyze KLF4 expression in sections after immunohistochemistry, immunoreactive system (IRS) was used to quantify the positively stained areas and intensity.

### Hydroxyproline Analysis

Total lung collagen levels were determined using Hydroxyproline Analysis Kit (Jiancheng, Nanjing, China). Briefly, a sample of lung tissue (30 mg) was mixed with 1 ml of hydrolysate and boiled for 20 min. One drop of indicator was added and pH was adjusted to 6.0–6.8. To a 3 ml sample of the digested pulmonary dilution, active carbon (about 30 mg) was added to make the supernatant clean and clear. After centrifugation, the supernatant was collected to test OD value at 550 nm by a spectrophotometer. Hydroxyproline concentrations were calculated from a standard curve of hydroxyproline.

## Materials

Bleomycin, recombinant human TGF-β1, and phalloidin were from Invitrogen (Carlsbad, CA, USA). Antibodies against E-cadherin, α-SMA, fibronectin, KLF4 and β-actin were from Santa-Cruz Biotechnology (Santa Cruz, CA, USA). Antibodies against Dvl-2 were from Cell Signaling Technology (Danvers, MA, USA). Antibodies against phosphorylated Smad2/3 were from Bioworld Technology (MN, USA). Antibodies against Smad2/3 were from Abcam (MA, USA, Beijing Biolink Biotehnology Co, Ltd).

### Statistical Analysis

Data are expressed as mean ± SEM. Multi-group comparisons were analyzed using Student’s *t*-test or one-way ANOVA. *P* < 0.05 was considered statistically significant. Non-quantitative results were representative of at least three independent experiments.

## Electronic supplementary material

Supplementary Material
