# Supplementary material for: Krüpple-like-factor 4 Attenuates Lung Fibrosis via Inhibiting Epithelial-mesenchymal Transition
Source: Sci Rep. 2017 Nov 20;7:15847. doi: 10.1038/s41598-017-14602-7 (PMC5696468; doi:10.1038/s41598-017-14602-7)
Supplement: Supplementary file 1 — Supplementary Material [file 41598_2017_14602_MOESM1_ESM.doc]

SUPPLEMENTARY MATERIALS

Krüpple-like-factor 4 Attenuates Lung Fibrosis via Inhibiting Epithelial-mesenchymal Transition

Lianjun Lin 1+, Qian Han 1+, Yan Xiong 2, Ting Li 2, Zhonghui Liu1, Huiying Xu1, Yanping Wu1, Nanping Wang3*, Xinmin Liu1*.


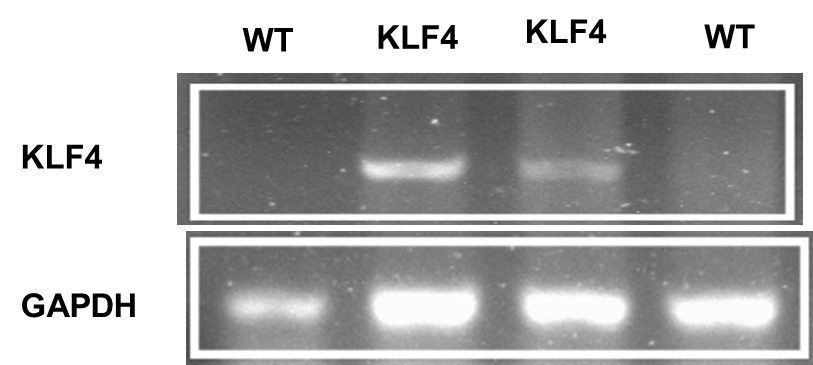


**Supplementary Figure 1. Characterization test of transgenic mice with overexpression of KLF4 with PCR.** Mice with overexpression of KLF4 were constructed by injection of plasmid DNA of KLF4 into zygote of FVB mouse following standard pronuclear injection by Cyagen Biosciences (CA, USA). Wild-type littermates were used as controls. The results of PCR showed that the expression of KLF4 in transgenic mice with overexpression of KLF4.


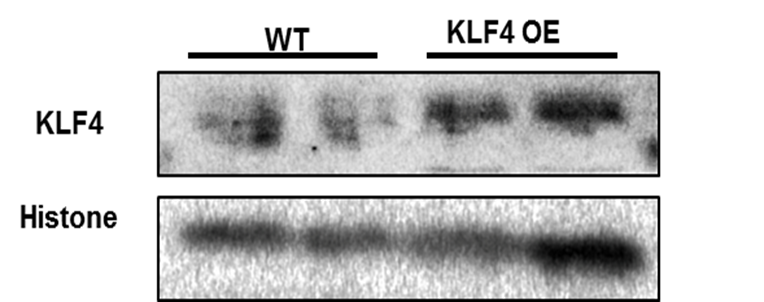


**Supplementary Figure 2. Characterization test of transgenic mice with overexpression of KLF4 with western blot.** Western blot showed the expression of KLF4 in lung tissues from transgenic mice with overexpression of KLF4 as well as in the wild type mice.

**a.**
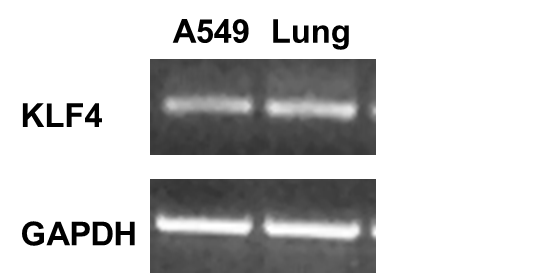
**b.**
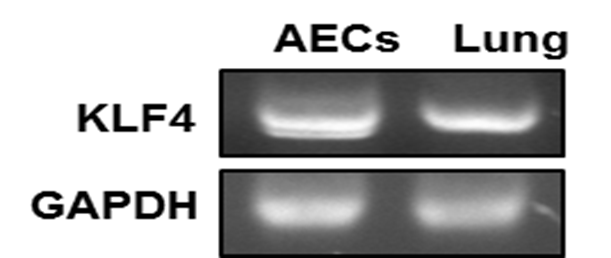


**Supplementary Figure 3. KLF4 mRNA level expression in Alveolar Epithelial Cells and lung tissues from mice with PCR. KLF4 mRNA extracted form A549 cells (a), human primary alveolar epithelial cells (b) and FVB mice lung tissue ( a and b).**

**a.**
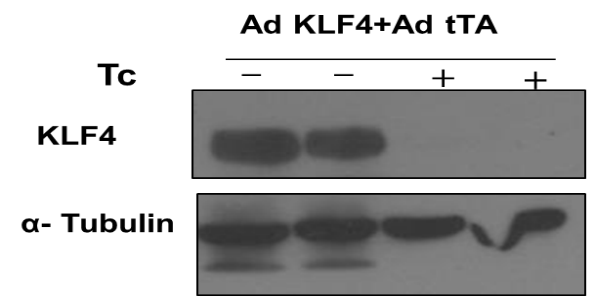
 **b.
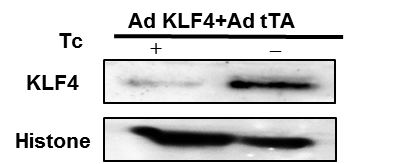
**

**Supplementary Figure 4. Adenovirus-mediated overexpression of KLF4 in Alveolar Epithelial Cells.** AECs were co-infected with AdKLF4 and AdtTA (20 MOI) and maintained in the medium with or without tetracycline (Tc; 0.1μg/mL). Nuclear protein lysates were immunoblotted with antibodies against KLF4 and α-tubulin or histone as an internal control. The results of western blot showed the expression of KLF4 in cells transfected with adenovirus-mediated overexpression of KLF4. Nuclear protein extracted from A549 cells (a) and human primary alveolar epithelial cells (b).

a.
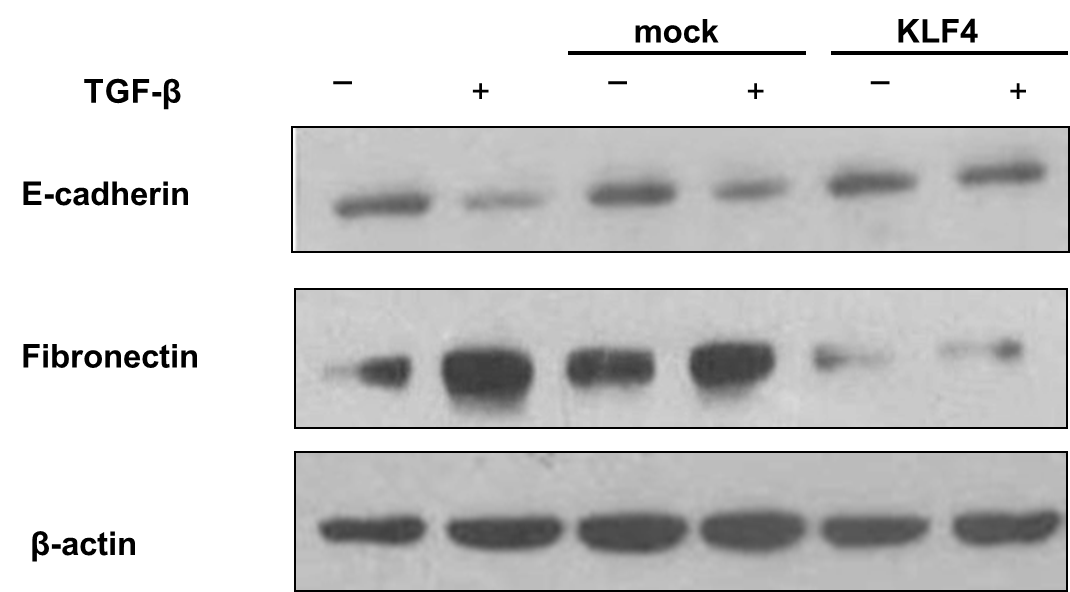


b.
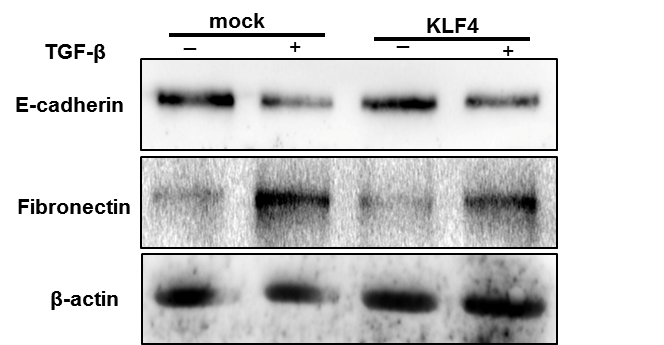


**Supplementary Figure 5. Overexpression of KLF4 attenuated phenotype markers expression during TGF-β1-induced EMT in Alveolar Epithelial Cells.** AECs were co-infected with AdKLF4 and AdtTA in the medium with or without tetracycline and then treated with or without TGF-β1 (5ng/mL) for 48 hours, and total proteins were immunoblotted with antibodies against E-cadherin and fibronectin. β-actin was used as loading control. Western blot showed expression of E-cadherin and fibronectin druing TGF-β1-induced EMT. Total protein was extracted from A549 cells (a) or human primary alveolar epithelial cells

a.
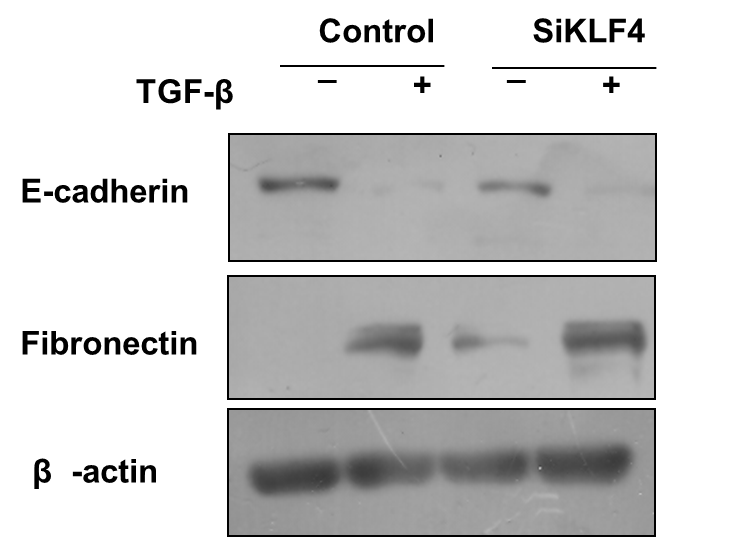


b.
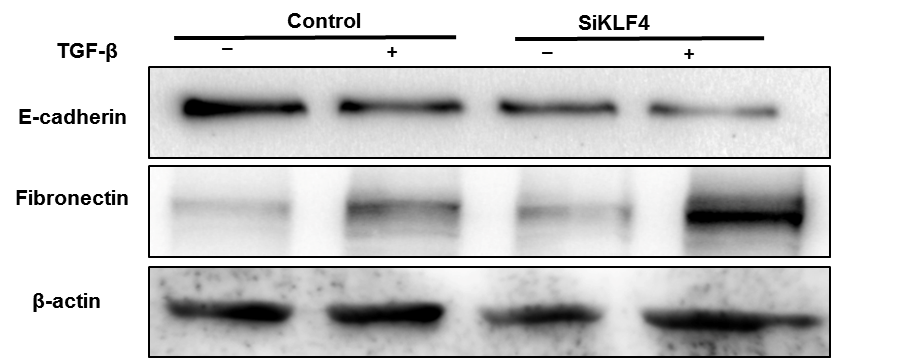


**Supplementary Figure 6. RNA interference of KLF4 enhanced phenotype markers expression during TGF-β1-induced EMT in Alveolar Epithelial Cells.** A549 cells (a) and human primary alveolar epithelial cells (b) were transfected with KLF4 siRNAs or control siRNA (100 nM) for 48 h. Total proteins were immunoblotted with antibodies against E-cadherin and fibronectin. β-actin was used as loading control. Western blot showed the change of E-cadherin and fibronectin during TGF-β1-induced EMT with knockdown of KLF4.

a.
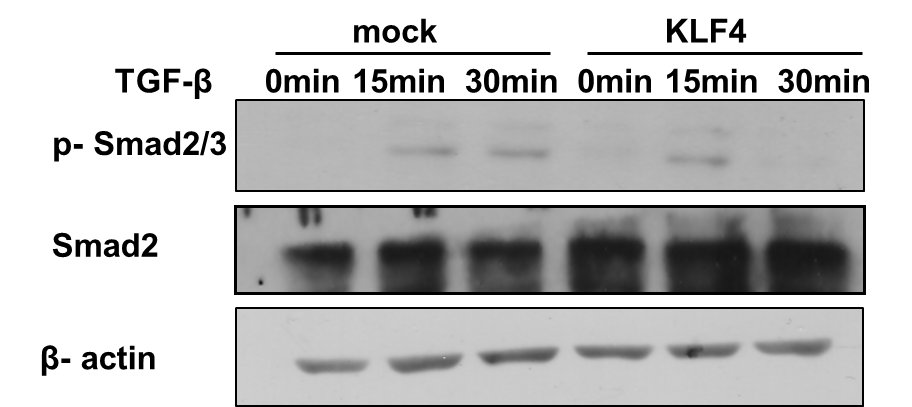


b.
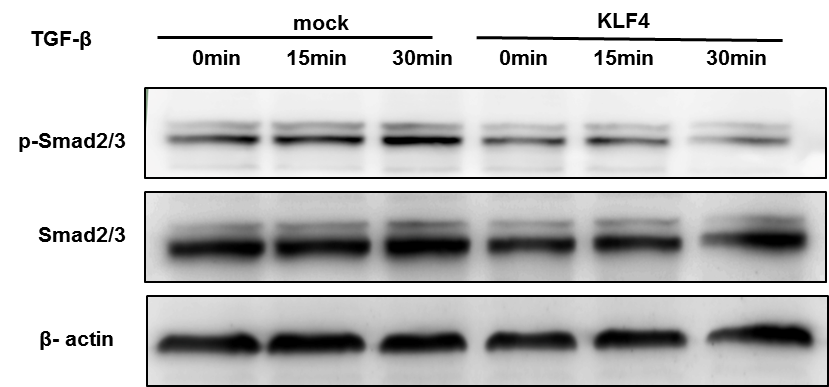


**Supplementary Figure 7. KLF4 inhibited phosphorylation of Smad2/3 signaling pathway during TGF-β1-induced EMT.** A549 cells (a) and human primary alveolar epithelial cells( b) were co-infected with AdKLF4 and AdtTA in the medium with or without tetracycline and then treated with or without TGF-β1 (5ng/mL) for indicated time. A Total protein lysate was immunoblotted with antibodies against Smad2/3, their phosphorylated forms, and β-actin. Results of western blot showed the change of phosphorylation of Smad2/3 with overexpression of KLF4 during TGF-β1-induced EMT.

a.
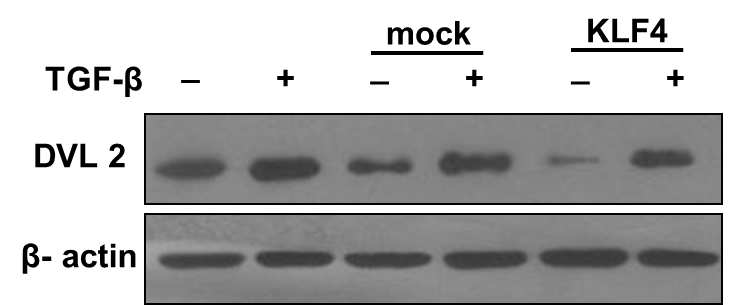


b.
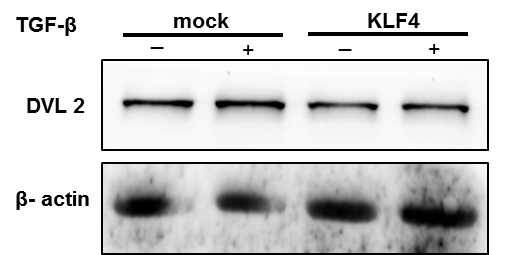


**Supplementary Figure 8. KLF4 inhibited Dvl Signaling pathway during TGF-β1-induced EMT.** A549 cells (a) and human primary alveolar epithelial cells (b) were co-infected with AdKLF4 and AdtTA in the medium with or without tetracycline and then treated with or without TGF-β1 (5ng/mL) for indicated time. A Total protein lysate was immunoblotted with antibodies against DVL-2 and β-actin. Results of western blot showed the expression of DVL-2 with overexpression of KLF4 during TGF-β1-induced EMT.

a.
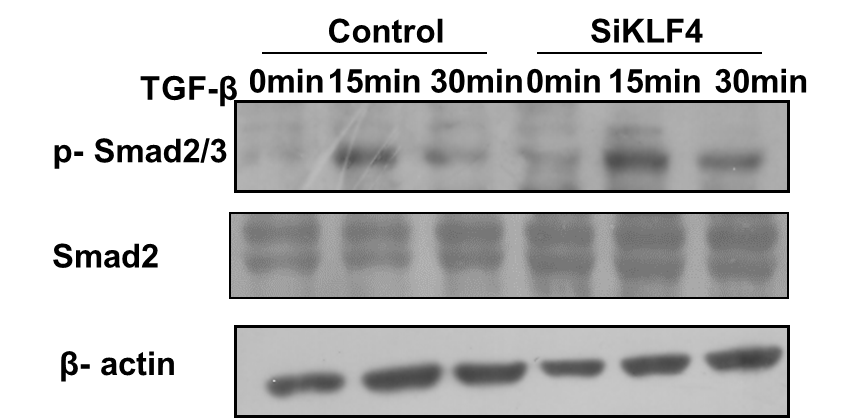


b.
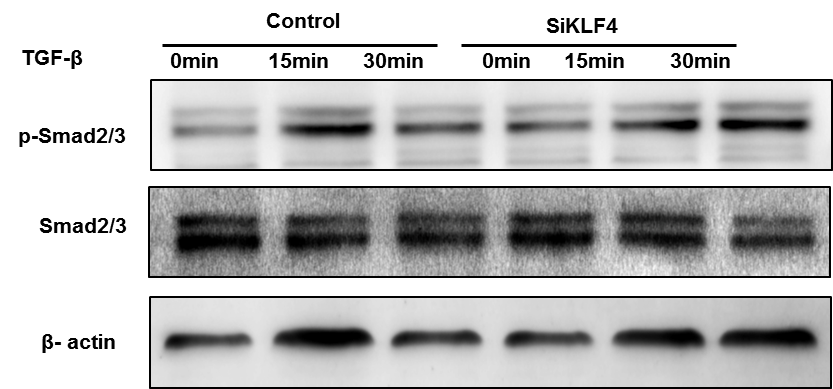


**Supplementary Figure 9. Knockdown of KLF4 potentiated TGF-β1-induced phosphorylation of Smad2/3.** AECs were transfected with KLF4 siRNAs or control siRNA (100 nM) and then were stimulated with TGF-β1 (5ng/mL) for indicated time. A Total protein lysate from A549 cells (a) and human primary alveolar epithelial cells (b) was immunoblotted with antibodies against Smad2/3, their phosphorylated forms, and β-actin. Results of western blot showed that the expression of phosphorylation of Smad2/3 with knockdown of KLF4 during TGF-β1-induced EMT.

a.
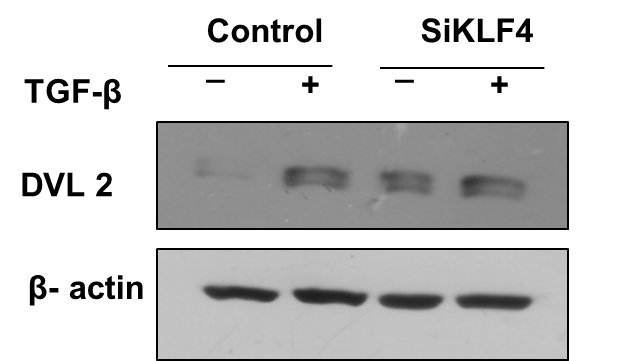


b.
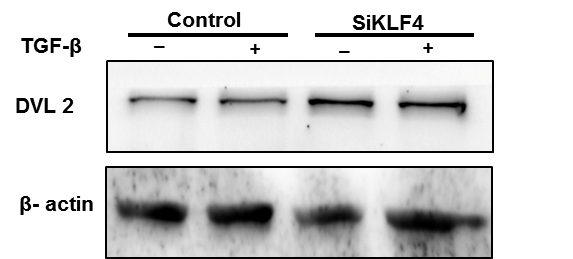


**Supplementary Figure 10. Knockdown of KLF4 potentiated DVL-2** **During TGF-β1-induced EMT.**AECs were transfected with KLF4 siRNAs or control siRNA (100 nM) and then were stimulated with TGF-β1 (5ng/mL) for indicated time. A Total protein lysate was immunoblotted with antibodies against DVL-2 and β-actin. Results of western blot showed the expression of DVL-2 with knockdown of KLF4 during TGF-β1-induced EMT. Total protein was extracted from A549 cells (a) and human primary alveolar epithelial cells.
